# Injectable Glycosaminoglycan-Based Cryogels from Well-Defined Microscale Templates for Local Growth Factor Delivery

**DOI:** 10.1021/acschemneuro.1c00005

**Published:** 2021-03-23

**Authors:** Ben Newland, Heike Newland, Francesca Lorenzi, Dimitri Eigel, Petra B. Welzel, Dieter Fischer, Wenxin Wang, Uwe Freudenberg, Anne Rosser, Carsten Werner

**Affiliations:** † Leibniz-Institut für Polymerforschung Dresden e.V., Max Bergmann Center of Biomaterials Dresden, Hohe Straße 6, D-01069 Dresden, Germany; ‡ School of Pharmacy and Pharmaceutical Sciences, Cardiff University, King Edward VII Avenue, Cardiff CF10 3NB, U.K.; § Dipartimento di Scienze Chimiche, Università degli Studi di Padova, via Francesco Marzolo, 135131 Padova, Italy; ∥ Charles Institute for Dermatology, University College Dublin, Dublin D04 V1W8, Ireland; ⊥ Brain Repair Group, School of Biosciences, Cardiff University, Cardiff CF10 3AX, U.K.; # Brain Repair And Intracranial Neurotherapeutics (BRAIN) Unit, Neuroscience and Mental Health Research Institute, Cardiff University, Hadyn Ellis Building, Maindy Road, Cardiff CF24 4HQ3, U.K.

**Keywords:** Heparin, photopolymerization, cryogel scaffold, nerve growth factor, sustained delivery, PC12 cells

## Abstract

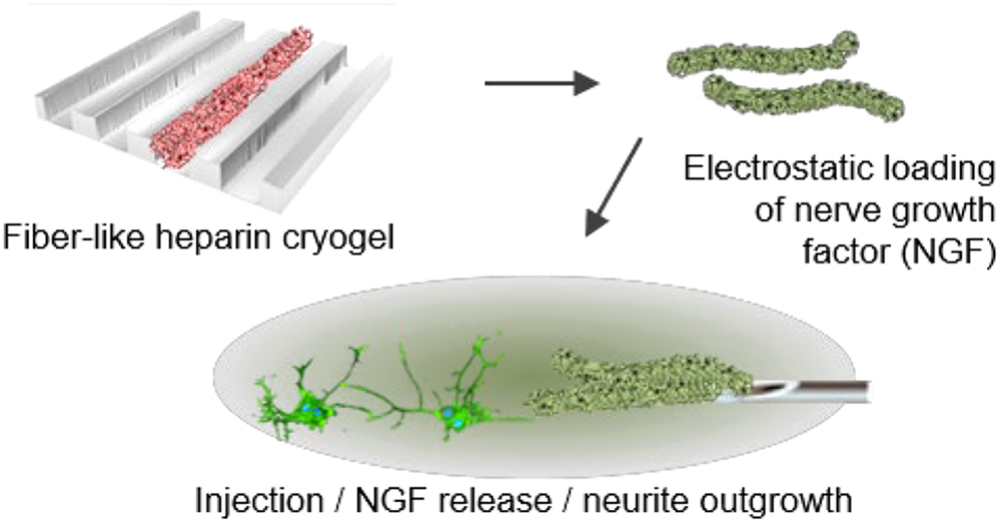

Glycosaminoglycan-based hydrogels hold great potential for applications in tissue engineering and regenerative medicine. By mimicking the natural extracellular matrix processes of growth factor binding and release, such hydrogels can be used as a sustained delivery device for growth factors. Since neural networks commonly follow well-defined, high-aspect-ratio paths through the central and peripheral nervous system, we sought to create a fiber-like, elongated growth factor delivery system. Cryogels, with networks formed at subzero temperatures, are well-suited for the creation of high-aspect-ratio biomaterials, because they have a macroporous structure making them mechanically robust (for ease of handling) yet soft and highly compressible (for interfacing with brain tissue). Unlike hydrogels, cryogels can be synthesized in advance of their use, stored with ease, and rehydrated quickly to their original shape. Herein, we use solvent-assisted microcontact molding to form sacrificial templates, in which we produced highly porous cryogel microscale scaffolds with a well-defined elongated shape via the photopolymerization of poly(ethylene glycol) diacrylate and maleimide-functionalized heparin. Dissolution of the template yielded cryogels that could load nerve growth factor (NGF) and release it over a period of 2 weeks, causing neurite outgrowth in PC12 cell cultures. This microscale template-assisted synthesis technique allows tight control over the cryogel scaffold dimensions for high reproducibility and ease of injection through fine gauge needles.

## Introduction

Growth factors have received much attention for applications in tissue engineering and regenerative medicine for their ability to affect cellular behavior and promote regeneration of damaged tissue. For example, in the field of neuroscience, several growth factors have been shown to be neuroprotective and are being investigated for therapeutic applications in Alzheimer’s disease,^1^ Parkinson’s disease,^2,3^ and spinal cord injury^4^ to name just a few. One major limitation of the use of growth factors is that they require repeat/frequent administrations due to their inherent short half-life (lack of serum stability).^2^ As such, pioneering research has developed protein pumps for the continual delivery, for example, of glial cell-line-derived neurotrophic factor to the brains of Parkinson’s disease patients to protect their dopaminergic neurons from the natural pathological progression of the disease.^2,5^

Hydrogels have been proposed as an alternative delivery system, releasing growth factors more slowly to the surrounding tissue than a bolus injection. To achieve sustained release of drugs and growth factors from biomaterials, our lab and others have incorporated heparin into the hydrogel structure.^6−9^ Heparin is known to bind a wide variety of growth factors with varying degrees of affinity, so it represents an ideal means of functionalizing hydrogels to increase loading and control the release of growth factors.

In order to incorporate heparin into hydrogels, the carboxyl groups on the heparin repeating unit can be reacted with amine groups on a cross-linker such as amine-terminated poly(ethylene glycol) (PEG) via an *N*-(3-(dimethylamino)propyl)-*N*′-ethylcarbodiimide (EDC)/*N*-hydroxysulfosuccinimide (sNHS)-assisted reaction.^10,11^ While this forms a stable covalent bond within biohybrid materials, the use of EDC and sNHS to assist the cross-linking process does not allow for *in situ* applications in the presence of cells due to toxicity issues. To overcome this drawback, the carboxyl groups of heparin can alternatively be modified to contain maleimide groups (again via EDC/NHS carboxyl–amine coupling). This maleimide-functionalized heparin can then be purified ready for use with thiol-containing compounds such as thiol-terminated multiarm PEG.^12^ This allows the addition of cells and growth factors to either of the gel components in their premixed state, which then forms a gel once combined and mixed. Despite much improvement in the synthesis and application of injectable hydrogels, they also carry an inherent drawback for use in tissue with little interstitial space (such as the brain), which is that they occupy almost the entire injection volume (nonmacroporous) and therefore create a dead space.^7^ Cell penetration into the gel is therefore subject to gel degradation. A further improvement over standard *in situ* forming hydrogels has been to fabricate microspheres that can be injected but annealed together to form a scaffold. In such cases, cells can grow throughout the spaces between the spheres (microporous scaffold) before degradation has taken place.^13,14^ However, in this case, the microspheres still occupy a large dead space until they are degraded.^13^

Macroporous scaffolds, with fine hydrogel struts and large interconnecting pores, offer a means of creating preformed, injectable materials without a large dead volume. Cryogelation represents a simple, but highly effective, method for producing such scaffolds, whereby the hydrogel components are frozen before/during network formation. Ice crystals form in the reaction mixture, thus cryoconcentrating the reactants between the ice crystals where they cross-link to form the hydrogel network. Once the ice is removed, a pore remains where the ice crystal was previously.^10,11^ The resulting materials, termed cryogelated hydrogels or cryogels, contain struts of hydrogel and large, interconnecting pores. Cryogel materials can be injected through needles with an inner diameter much smaller that the width of the material, since it compresses like a sponge and re-expands to its original volume and shape once through the needle.^15−17^

**Figure 1:**
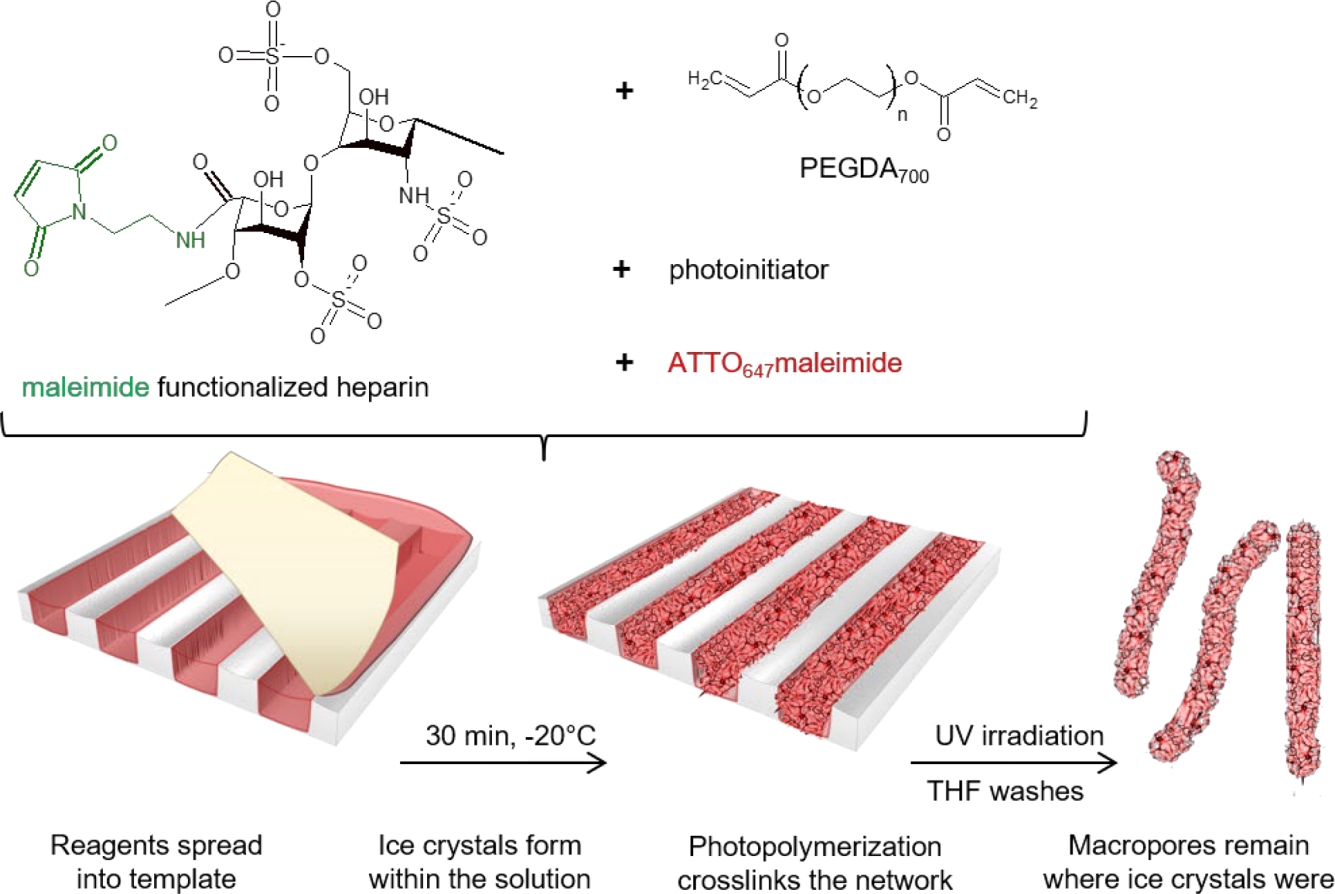
Schematic depiction of the cryogel synthesis process, showing the maleimide-functionalized heparin and poly(ethylene glycol) diacrylate, the process of template filling, subzero polymerization, thawing, and template removal.

Cryogels are typically quite robust, exhibiting good tough mechanical properties, making them easy to handle for applications in *ex vivo* slice culture^18,19^ and local delivery.^20^ However, in addition, their ability to be compressed through an injection cannula is of interest for use in the brain,^21^ where surgical intervention is limited. Unlike spherical microscale cryogels (termed microcarriers) developed for cell culture^22^/cell transplantation,^11^ this work aimed to produce high-aspect-ratio, fiber-like cryogels as a step toward bridging anatomically connected regions of the brain or nervous system. For example, using an extrusion style of injection,^23^ one could envisage laying a line of fibers along the nigrostriatal pathway affected by Parkinson’s disease in a manner similar to “bridge grafts”.^24^ We hypothesized that strict size control could be achieved by forming the cryogels within well-defined sacrificial templates (Figure 1). Templates containing channels, micrometers in width and depth but millimeters in length, would allow the formation of long cryogels that could still be injected through fine gauge needles. We herein show that photoinitiated polymerization can be used as a quick and convenient method to cross-link maleimide-modified heparin and PEGDA after the precursor solution has been frozen, forming biohybrid cryogels that can load and release NGF.

## Results and Discussion

**Figure 2:**
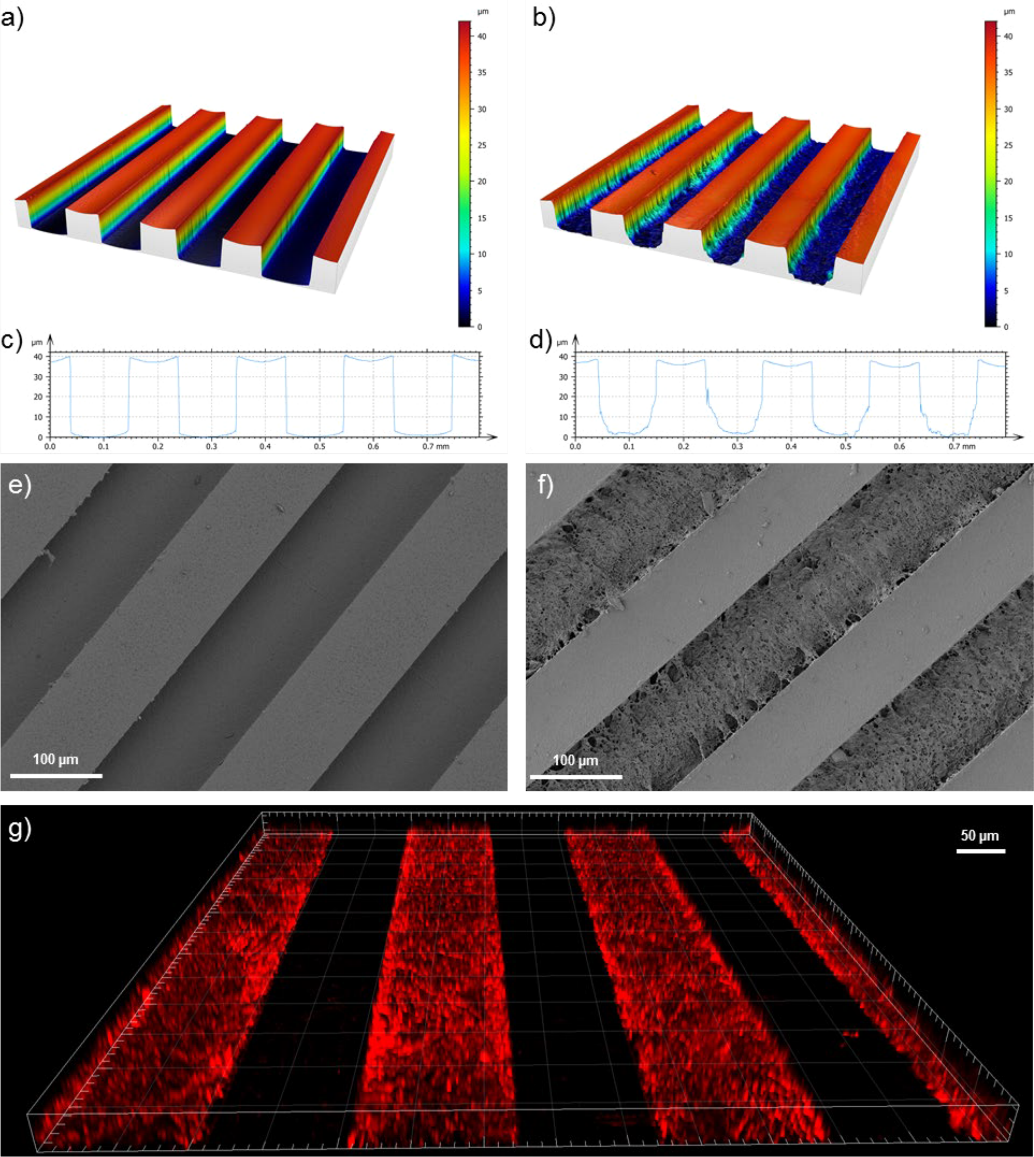
Analysis of the templates containing channels 40 μm deep and 100 μm wide. Multipinhole confocal microscopy analysis of the polystyrene template empty (a) or filled post polymerization (b) with corresponding profile analysis (c,d) of the empty and filled templates, respectively. Scanning electron microscope image of an empty template (e) and a filled template (f). Confocal laser fluorescence microscope image (g) of the filled template in the PBS hydrated state showing lines of macroporous cryogel scaffolds.

Microstructured polystyrene templates were produced by solvent-assisted microcontact molding using PDMS stamps that were created from three different silicon masters. These templates were 10 × 10 mm with channels of 70, 100, or 150 μm in width and a depth of 40 μm as determined by multipinhole confocal microscopy (Figure 2a,c,e and Supporting Information, Figures S1–S3). The precursor solution containing maleimide-functionalized heparin, poly(ethylene glycol) diacrylate (PEGDA), the photoinitiator, and the fluorescent label was added into the plasma-treated template as depicted in Figure 1. Freezing the solution-filled template at −20 °C for 30 min allowed ice crystal formation in the solution, and subsequent cross-linking via the application of UV light (3 min) resulted in a cross-linked polymer network that could be thawed and then removed from the template via THF dissolution.

Multipinhole confocal microscopy and scanning electron microscopy of the cryogels within the template revealed that while dehydrated they have a compacted structure that spans the length and width of the template but not the full height/depth (Figure 2b,d,f). Along the centerline of the channel, the cryogel is quite flattened to the template surface as shown by the profile measurements in Figure 2d and Supporting Information, Figures S1b, S2b, and S3b. However, once the structure is hydrated in PBS and imaged by fluorescence confocal microscopy, the cryogels swell approximately to the full height/depth without the appearance of a meniscus-like shape (Figure 2g).

It should be noted here that the wavelength of UV light chosen for the photoinitiation (366 nm) did not photobleach the samples, since the far-red (610 nm) ATTO dye was chosen for the cryogel functionalization. Although we have not tried, one could envisage that commonly used dyes with lower excitation wavelengths (e.g., 305–488 nm) may get photobleached during the cross-linking process. A low-wavelength UV lamp and a corresponding photoinitiator such as 2-hydroxy-2-methylpropiophenone^25^ (large absorption band at 245 nm) would allow the use of green and near-red fluorescent labels.

Raman spectroscopy analysis of unlabeled cryogels in comparison to the free reagents (PEGDA and heparin maleimide) shows that the photoinitiated cross-linking reactions indeed exhaust the acrylate and maleimide groups (Supporting Information, Figures S4 and S5). The peak due to the maleimide in heparin maleimide (C=O: 1774 cm^–1^) is reduced. Likewise, the characteristic peaks due to the acrylate in PEGDA (C=C: 1640 cm^–1^, C=O: 1723 cm^–1^) are completely reduced and blue-shifted (1732 cm^–1^), which occurs as a result of the radical polymerization of PEGDA. Additionally, the presence of the characteristic sulfate group (R–O–SO_3_^–^) vibrations of heparin (890, 1070 cm^–1^) in the spectrum, show its successful incorporation into the cryogel network. However, these data cannot rule out the possibility that we have formed two separate interpenetrating networks of PEGDA and heparin.

Since heparin is a highly negatively charged molecule due to its sulfate groups, we sought to analyze the distribution of the heparin throughout the cryogel by using a positively charged fluorescent molecule (doxorubicin) that can electrostatically bind to heparin but not PEG.^26^Supporting Figure S6 shows that fluorescence of labeled heparin shown in green largely overlaps with that of bound doxorubicin (red), showing that heparin is distributed throughout the cryogel, though there are areas of slightly higher intensity than others. A good correlation between the two fluorescent signals was observed as shown by a Pearson’s R value of 0.74 (where +1 shows perfect correlation, 0 shows no correlation, and −1 shows anticorrelation).

**Figure 3:**
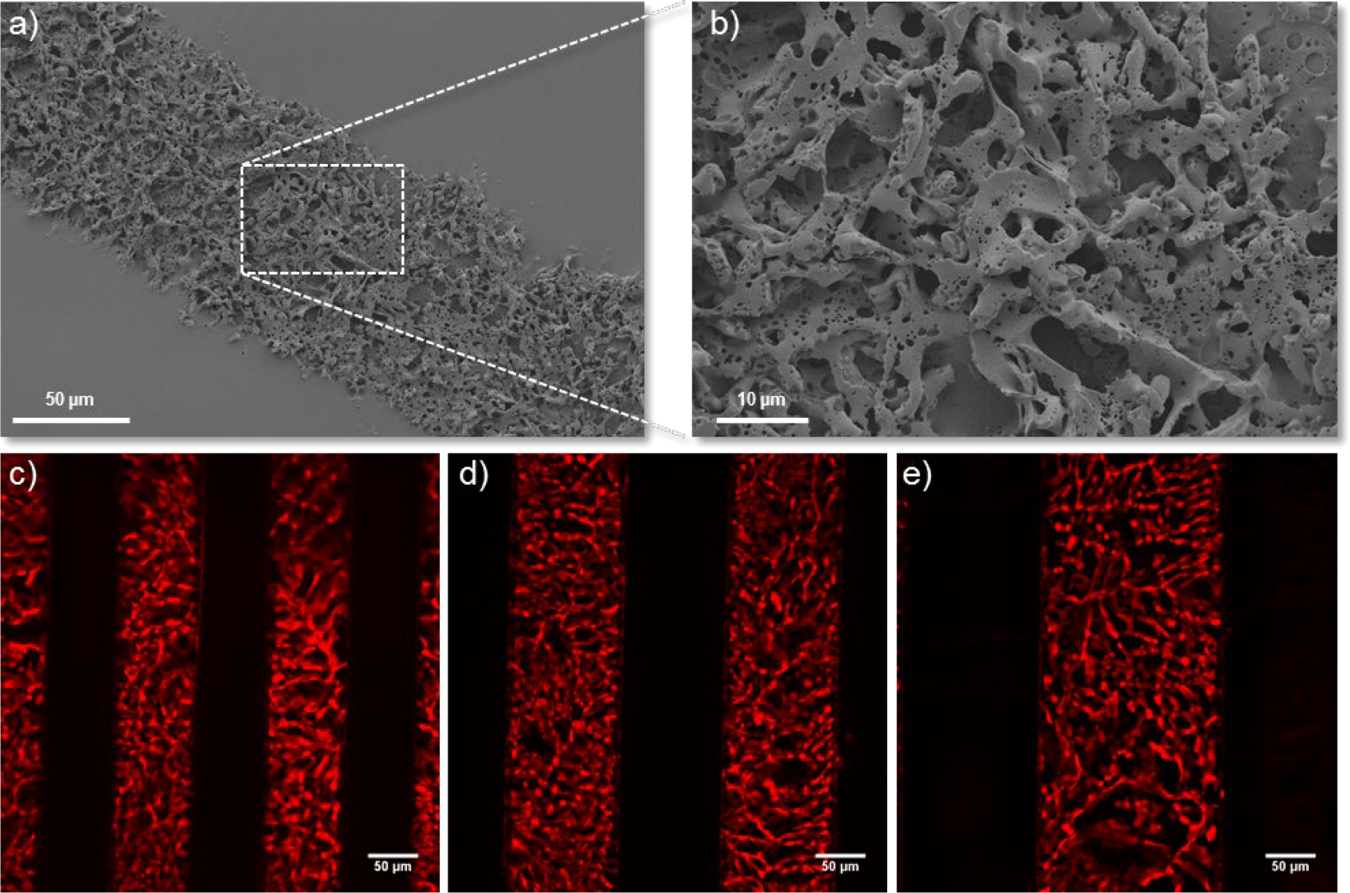
Scanning electron microscopy analysis of a heparin-based cryogel scaffold in its dehydrated state (a,b) showing the porous nature of the scaffold. Fluorescence microscopy analysis of ATTO-610-labeled cryogel scaffolds in their hydrated state, which had been synthesized in 70 μm channels (c), 100 μm channels (d), and 150 μm channels (e) showing the high precision control over scaffold dimensions.

Ice crystal formation, prior to polymer network formation, is a useful means to form macroporous structures in hydrogel materials. Using ice as a porogen has the advantages of being easy to wash out, with no porogen additives needed, and the pore size can be varied by changing the rate of cooling.^27^ However, the pore size distribution is rather large, which could be disadvantageous if a fixed pore size is required. Scanning electron microscopy of a dehydrated scaffold (Figure 3a,b and Supporting Information, Figure S7) shows the macroporous structure of the cryogel. However, we evaluated the pore size distribution in the more physiologically relevant hydrated state using confocal laser scanning microscopy (CLSM). Supporting Figure S8 illustrates the pore size distribution based on the CLSM measurements for these microscale cryogel scaffolds, with an average pore diameter of 9.9 μm. By varying the dimensions of the template, the size of the resulting cryogel can be precisely controlled (Figure 3). Three different templates were used with channel widths of 70 μm (Figure 3c), 100 μm (Figure 3d), and 150 μm (Figure 3e). The μ-contact printing method of template formation allows well-defined and reproducible cryogel shapes to be formed without the need for punching out shapes form a bulk cryogel scaffold.^21^ While cryogels from millimeter-sized molds have been injected through a 16 gauge needle,^15,16^ herein we aimed to create high-aspect-ratio materials with precise microscale widths. The macroporous structure and small width of the cryogels formed herein allow them to be injected through a much smaller needle (30 gauge) (Supporting Information, Figure S9). Additionally, in principle, one can envisage the use of this technique for disc or other shaped materials as defined by the silicon master and corresponding template.

Design flexibility in terms of the size and shape of the cryogel scaffolds broads the range of potential end-user applications. For example, when using cylinder-shaped PEGDA cryogels to deliver a demyelinating agent to central nervous system slice cultures, smaller cryogels were used for the spinal cord sections than the larger cortical sections.^18^ Where either whole neural pathways or large brain areas are to be targeted, high-aspect-ratio scaffolds may be able to bridge across regions. Although application of elongated cryogels to *ex vivo* coslice models (such as the Parkinson’s disease relevant nigrostriatal pathway^28^) has recently been achieved,^29^*in vivo* application would likely require sophisticated injection/extrusion techniques.^23^

**Figure 4:**
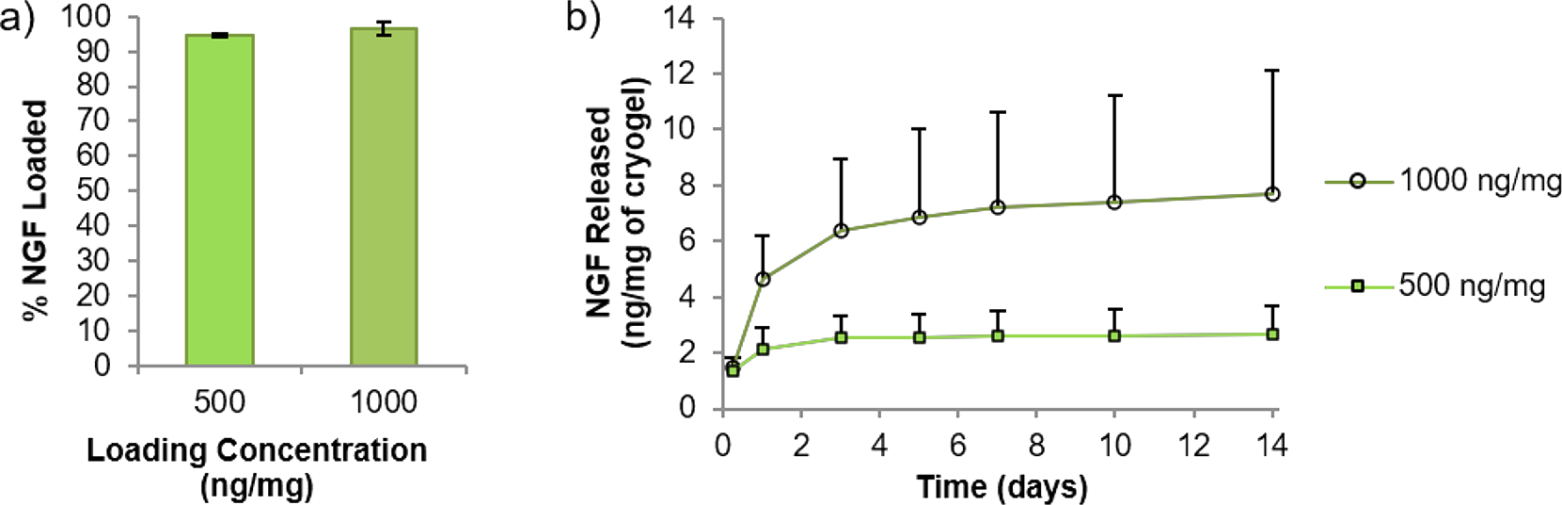
Loading and release of nerve growth factor (NGF) from the heparin-based microscale cryogel scaffolds. (a) shows that almost all of the NGF was loaded from the loading solutions (500 ng/mg of cryogel or 1000 ng/mg of cryogel) to the cryogel scaffold and was slowly released (b) over a period of 2 weeks (longest time analyzed) (*n* = 3) (error bars represent the cumulative value of standard deviation).

The use of the highly sulfated glycosaminoglycan heparin in biomaterials allows noncovalent, electrostatic/reversible binding of growth factors followed by sustained release over extended periods of time.^7,11,30^ Herein, the uptake of NGF to the cryogels was assessed at two different concentrations of either 500 or 1000 ng of NGF per 1 mg of cryogel (Figure 4). These amounts of NGF were chosen, because we have previously shown that heparin-based microcarriers release less than 3% of their growth factor payload,^11^ thus requiring a high loading amount to see a biological effect in a short time frame. While the literature cites NGF as only having low/moderate affinity toward heparin,^31,32^ the cryogels took up almost all the NGF from both concentrations of the solution (94.5% for 500 ng/mg and 96.6% for 1000 ng/mg).

The release of bound NGF into PBS was measured over a period of 2 weeks, and the cumulative release profile is plotted in Figure 4b. This shows that for the cryogels loaded with 500 ng of NGF per 1 mg of cryogel, a plateau was reached after 3 days, whereas for the sample loaded with more NGF (1000 ng/mg), the release continues slowly from 3 to 14 days. The released NGF after 2 weeks represents 0.5 and 0.8% of the total NGF loaded to the cryogels (Supporting Information, Figure S10), which is approximately in line with results previously obtained from heparin-based microcarriers.^11^ The high amount and rapid loading of NGF, together with the slow release, indicate that NGF has sufficient affinity toward heparin to allow for sustained delivery from these heparin-based cryogels.

**Figure 5:**
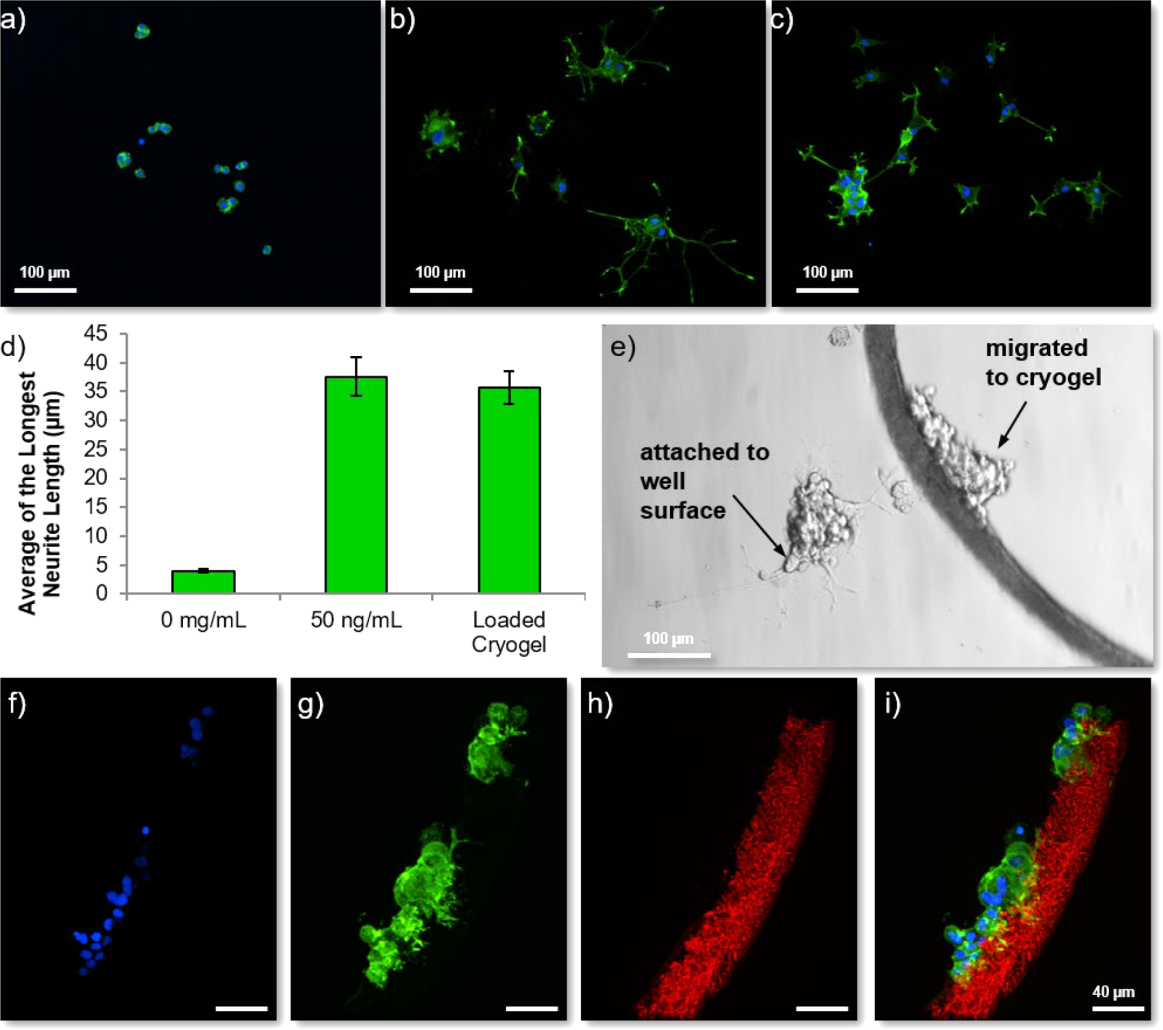
Analysis of the bioactivity of the released NGF. Fluorescence microscopy analysis of PC12 cells (blue = nuclear stain Hoechst, green = anti-βIII-tubulin staining) grown in the absence of NGF (a) show that almost no neurites can be observed, whereas the addition of NGF to the culture medium at a concentration of 50 ng/mL (b) causes long neurites to extend from the cell bodies. PC12 cells cultured in the presence of NGF-loaded cryogels (c) also show neurite extensions as quantified by analysis of the longest neurite lengths per cell (d) (error bars represent ± standard error of the mean (100 neurites measured)). An example light microscope image (e) of the PC12 cells cultured in the presence of NGF-loaded cryogels showing some cells adherent to the tissue culture plastic and some attached to the cryogel. (f–i) Fluorescence microscopy of the cells attached to the ATTO-610-labeled cryogel (red) as observed via a Hoechst nuclear stain (blue) and anti-βIII-tubulin staining (green) showing small projections from the cells into the cryogel structure.

To analyze whether NGF released from the cryogels is bioactive, cryogels were loaded with NGF at a higher concentration (1000 ng/mg of cryogel) and placed into a culture of PC12 cells (Figure 5). Despite being derived from a pheochromocytoma of the rat adrenal medulla, PC12 cells are commonly used as a model neuron, as they produce neurites when exposed to NGF.^33^ As with such immortalized cell types, care must be paid when drawing conclusions from their use; however, herein they were used as a means of assessing the bioactivity of the released growth factor.

Figure 5 shows PC12 cells stained with antibodies against βIII-tubulin (also referred to as Tuj-1), a protein associated with microtubule stability in neurons. Cells grown in the absence of NGF showed the typical rounded morphology (Figure 5a), but cells treated with NGF added directly to the medium at a concentration of 50 ng/mL (Figure 5b) for 7 days had neurite extensions. In addition, cells exposed to/in contact with NGF-loaded cryogels also extended neurites as shown in Figure 5c. Analysis of the length of the longest neurites for each cell showed that there was no significant difference between those cells treated with the NGF protein in solution and those exposed to the NGF-loaded cryogels (Figure 5d), despite *in vitro* release being minimal. We had previously questioned the therapeutic utility of such a delivery device when such a little amount of NGF is released, though we would predict higher release rates *in vivo* due to competitive binding of other serum/extracellular proteins displacing NGF. Nevertheless, these results indicate that NGF-loaded scaffolds can cause cellular differentiation, at least in the *in vitro* PC12 cell model.

**Figure 6:**
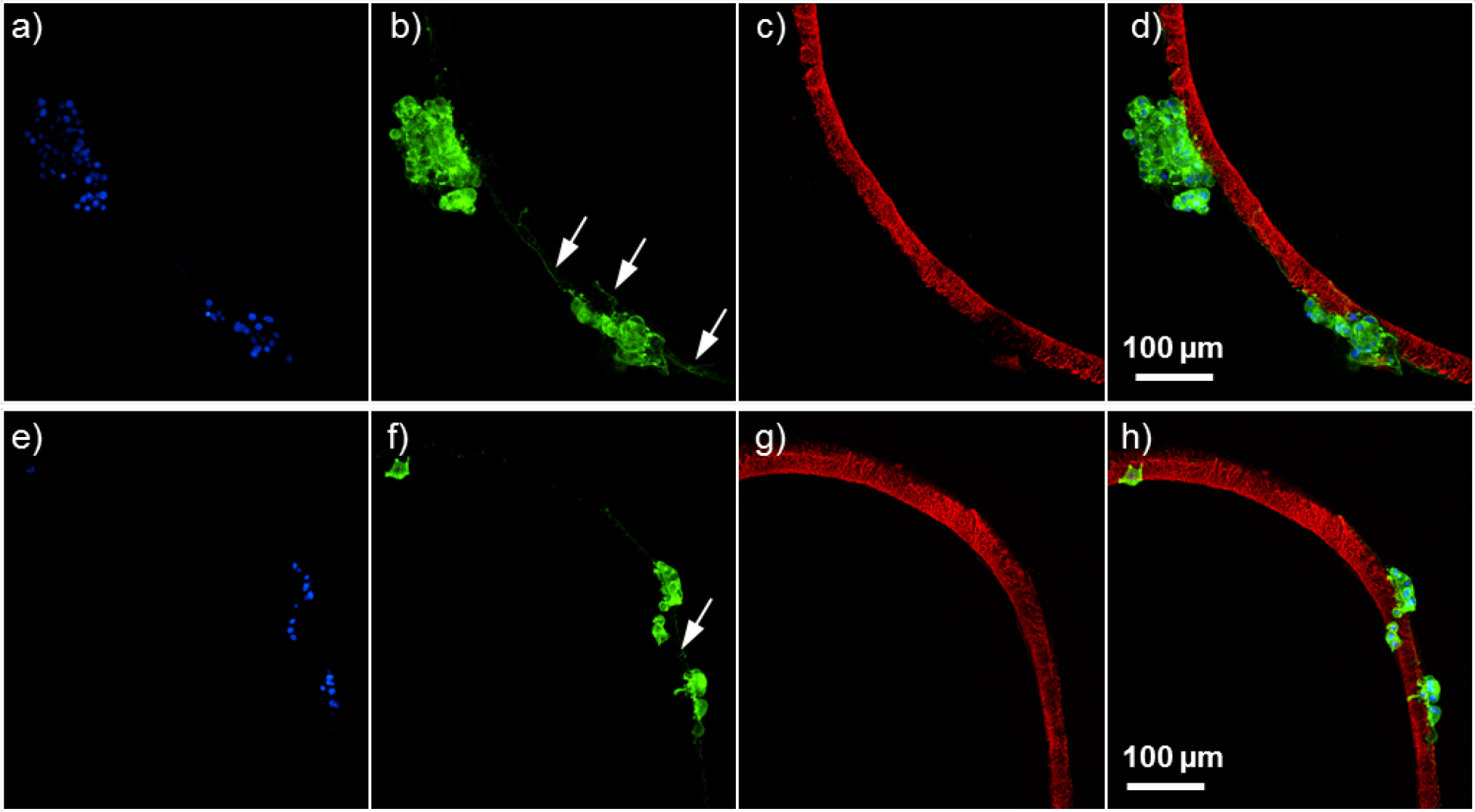
PC12 cells extend neurites along NGF-loaded cryogel scaffolds. Fluorescence microscopy of PC12 cells attached to the ATTO-647-labeled cryogel (c,g, red) as observed via Hoechst nuclear stain (a,e, blue) and anti-βIII-tubulin staining (b,f, green) with the channels merged (d,h) showing neurites from the cells projecting along the cryogel structure (indicated by white arrows).

Interestingly, we observed that quite a number of the cells had migrated from the well bottom (where they had been allowed to adhere before beginning the experiment) onto the cryogels. An example light microscope image (Figure 5e) shows cells that are adhered to the well plate and cells that are attached to one of the cryogels in culture. This is intriguing, since the cryogels have not been functionalized to improve cell adhesion (e.g., coating with poly-D-lysine (PDL) or addition of cell adhesion ligands). Furthermore, fluorescent microscopy analysis of the cryogels that had cells migrated onto them showed that while the majority of cells stayed in a round form, a few had produced neurites that penetrated into the porous structure of the cryogel (Figure 5f–i). Figure 6 shows that some of the PC12 cells even extended neurites along the outmost surface of the NGF-loaded cryogels. For applications in directing nerve regrowth such as spinal cord injury or peripheral nerve injury, one could consider functionalizing the scaffold with a cell adhesion ligand such as arginine–glycine–aspartate (RGD),^34^ which allows multiple cell types to adhere to cryogel surfaces^22^ as this may further improve neurite extensions through these NGF-loaded matrices.

These loading, release, and cell culture studies show that bioactive NGF can be released up to at least 2 weeks; however, since only a small proportion of the NGF is released, the cryogels effectively retain a large amount of the growth factor. If faster NGF release would be required, the heparin could first be subjected to selective desulfation procedures, to remove some of the sulfate groups allowing quicker protein release.^35−37^

Although we have mentioned some uses of cryogel scaffolds in *ex vivo* slice culture, they may also hold potential for neurotherapeutic delivery to the brain. There are a range of growth factors that give rise to neuroprotection or have regenerative effects, so improving the spatial and temporal control over their delivery is of interest.^38^ Unlike the spinal cord, where macroscopic nerve guiding conduits have been implemented to promote and guide axonal growth through the neuroinhibitory glial scar,^39−41^ restricted surgical access to the brain requires the use of injectable delivery devices. Studies have utilized growth factor secreting cells encapsulated in a hydrogel^42,43^ as a means of delivering growth factors to the brain with the clear advantage of continual production for as long as the grafted cells remain viable. The injection of these cells has typically been unifocal, resulting in approximately even delivery to the regions surrounding the graft.^43^ However, there are instances where guiding neural regeneration in the brain may be of therapeutic interest. For example, in Parkinson’s disease where dopaminergic neurons die back from the striatum (cell terminals) to the substantia nigra (where the cell bodies reside), researchers have proposed using a “nigrostriatal bridge” to promote regeneration of neuronal networks following cell transplantation to the substantia nigra.^24,44,45^

One of these early studies used fibroblast-growth-factor-4-transfected RN-22 schwannoma cells injected so as to form a line between a dopaminergic cell graft in the substantia nigra and the striatum.^24,46^ A bridge graft up to 1.5 cm was shown to contain many axons from the dopaminergic graft growing toward, and projecting into, the striatum.^46^ There has not been extensive study of using materials for such a purpose, although the host response to high-aspect hydrogels placed in the nigrostriatal pathway has been analyzed.^47^ Elongated, or fiber-like, cryogel scaffolds represent an alternative to the more abundantly studied classical hydrogel. The porous nature not only avoids occupation of a large dead space in the brain (as neurites can grow into the scaffold) but also allows a predefined, preformed, but compressible shape to be injected. This work shown herein shows the first steps toward injectable cryogel materials that could be used for a “bridging” application.

In summary, we have photopolymerized PEGDA with maleimide-modified heparin within a well-defined polystyrene template. The reactants were first frozen so that the ice crystals that formed subsequently left behind pores in the structure. The photopolymerization occurred successfully at a subzero temperature to yield porous cryogel scaffolds of exact diameter and depth in a variable microstructured shape. The use of UV light did not photobleach the fluorescent ATTO 610 label that was used to functionalize the scaffold. The scaffolds could be loaded with large amounts of NGF, and a small percentage of this could be released over a period of 2 weeks causing neurite outgrowth in a PC12 cell culture model. In contrast to hydrogels, these microscale cryogels exhibited excellent mechanical properties and could retain their shape and integrity after injection through a needle routinely used for intracranial delivery. These data show that cryogels can be synthesized on the microscale in a precise manner, opening the door for further investigation into their use as a delivery device for growth factors to the brain or spinal cord.

## Materials and Methods

### Fabrication of the Sacrificial Polystyrene Template

Polystyrene templates were produced via a similar method as reported by Müller et al.^48^ Silicon masters featuring channels 10 mm in length, 40 μm in depth, and either 70, 100, or 150 μm in width were created using photolithographic etching (GeSiM, Germany). From these silicon masters, a poly(dimethylsiloxane) silicone (PDMS) (Sylgard 184 silicone elastomer kit, Dow Corning, Germany) stamp (i.e., inverse of the master features) was created within polycarbonate holders (GeSiM) by curing the PDMS (10:1 curing agent to prepolymer) at 80 °C for 2 h. The PDMS stamp was loaded to a μ-contact printer (GeSiM) and wetted with 30 μL of ethyl acetate (Sigma, Germany). The stamp was then immediately lowered to a fixed position, where it was pressed onto a flat clear polystyrene sheet with a thickness of 40 μm (Evergreen Scale Models, USA). After 15 min at 21 °C, the PDMS stamp was raised, and the microstructured polystyrene sheet was removed.

### Template Characterization

The dimensions and the topography of the polystyrene template were analyzed via multipinhole confocal microscopy utilizing a μsurf explorer (NanoFocus AG, Germany) equipped with a 10× objective (Olympus, Germany). 3D height maps were generated using μSoft analysis software (NanoFocus AG). In addition, the templates were characterized by scanning electron microscopy (SEM). The template was adhered to specimen stubs via carbon adhesive and sputter-coated with gold for 40 s (BALZERS SCD 050 Sputter Coater, Germany). Imaging was performed using a XL30 ESEM-FEG microscope (Philips, Netherlands) in high-vacuum mode using accelerating voltages of 10 kV.

### Cryogel Synthesis

Cryogels were fabricated within the channels of polystyrene templates, where each sheet could produce between 30 and 70 cryogels depending on the channel width used. In order to incorporate heparin into the cryogel structure, heparin that had been modified to contain six maleimide groups per heparin molecule was used (henceforth termed heparin maleimide). The modification of the heparin by linking maleimide amine to the heparins’ carboxylic acid groups using 1-ethyl-3-(3-(dimethylamino)propyl)carbodiimide (EDC) and *N*-hydroxysulfo-succinimide (sNHS) was reported previously.^12^

The polystyrene template was first treated with low-pressure oxygen plasma (PDC-002, Harrick Plasma, USA) for 60 s to increase the wettability of the template. A precursor solution was prepared fresh prior to use, containing polyethylene glycol diacrylate (PEGDA) of an average molecular weight of 700 Da (Sigma) at 0.1 g/mL, heparin maleimide at 0.1 g/mL (molar ratio 1:0.05 PEGDA/heparin maleimide), lithium phenyl-2,4,6-trimethylbenzoylphosphinate (LAP) (0.2 mg/mL) as a photoinitiator, and ATTO 610 maleimide (ATTO-TEC, Germany) (0.1% v/v) as a fluorescent dye. A 4 μL aliquot of this solution was added to the template, which filled the channels. Excess was removed by drawing a polystyrene sheet across the top of the template (see Figure 1). The template was then placed at −20 °C for 30 min before being exposed to UV light (8 W hand lamp, wavelength 254 nm, Benda, Wiesloch) for 3 min while still at −20 °C.

The cryogels were left to thaw, and since the polystyrene templates are clear, the cryogels were also visualized at this stage via light microscopy and fluorescence microscopy. SEM analysis was also performed for cryogels left within the template.

The microscale cryogels were harvested from their template by dissolving the template in 1 mL of tetrahydrofuran (THF) (Sigma). After 2 min of gentle shaking, the cryogels parted from the template and were transferred by pipet to a preweighed centrifuge tube, where they were washed four times with THF (to remove dissolved polystyrene), three times with water (to remove THF), and twice with absolute ethanol (Sigma) and left to dry in a vacuum oven. The filled centrifuge tubes were then reweighed to obtain the dry mass of the cryogels.

### Cryogel Characterization

Spinning disc confocal laser microscopy (SPCLM) was used to visualize the cryogels and measure the pore size in the hydrated state. A Dragonfly SPCLM (Andor Technology, U.K.) mounted on an Nikon Ti-E inverted microscope was used with a 637 nm laser diode, and images were taken with either a 10× magnification objective (CFI Plan Apo Lambda, Nikon) or a 20× magnification objective (CFI Plan Apo Lambda, Nikon). Pore sizes were analyzed from a single *z*-plane by measuring 100 pores in both the *x*- and *y*-planes using ImageJ software (NIH). Where a stack of *z*-planes was imaged, a *z*-distance of 0.5 μm was used. To confirm the incorporation of heparin maleimide, cryogels were synthesized as described above but without the inclusion of the fluorescent label. Raman spectroscopy analysis of the PEGDA monomer, heparin maleimide, cross-linked PEGDA, and the PEG/heparin cryogels was performed using the Confocal Raman Microscope alpha 300 R (WITec GmbH, Germany) equipped with a laser with an excitation wavelength of 785 nm and a laser power of 500 μW. Samples were measured with a 20× objective (Zeiss) and an integration time of 0.5 s for a single scan in the wavelength region from 660 to 1850 cm^–1^. For each spectrum, 200 accumulations were performed.

### Growth Factor Loading and Release Analysis

Cryogels were dried on a 0.4 μm hanging cell culture insert (*n* = 3 inserts of cryogels) and weighed. The dried cryogels were resuspended in nerve growth factor (human β-NGF), PeproTech, Germany) dissolved in phosphate buffered saline (PBS) at a concentration of 5 ng/mL (termed the loading solution). The amount of loading solution added was calculated to accomplish a NGF to cryogel weight ratio of either 500 ng/mg of cryogel or 1000 ng/mg of cryogel (*n* = 3). In addition, three culture inserts containing no cryogels (termed “empty” inserts) were filled with 300 μL of 5 ng/mL of NGF to act as a control when determining the percentage of NGF loaded to the cryogels. The cryogels in the loading solution and the control loading solution were left for 72 h at 37 °C in a sealed well plate. This loading solution was then removed (by centrifuging the plate containing the insets at 1000 rpm for 1 min (2–16KL Sigma Centrifuges, Germany) and frozen at −80 °C for subsequent analysis. The cryogels were washed with 600 μL of PBS per culture insert, and the wash solution was also retained for analysis. The removed wash solution was replaced with 600 μL of PBS. The cryogels were incubated at 37 °C, and at each time point (1, 3, 5, 7, 10, and 14 days), the solution was centrifuged through the culture insert, removed, and stored at −80 °C and replaced by 600 μL of fresh PBS. After 14 days, all stored samples were defrosted, and 100 μL of each sample was analyzed using an NGF enzyme-linked immunosorbent assay (ELISA) (human β-NGF, DuoSet, R&D Systems, Germany) according to the manufacturer’s instructions. Dilutions were made where necessary to ensure that the sample concentrations fell within standard curve obtained via the provided standard protein. The percentage of loaded NGF was determined by subtracting the loading solution and wash solution from the value of NGF from the “empty” inserts. The results were plotted as cumulative release with the standard deviation for each time point cumulated.

### PC12 Cell Culture and Neurite Outgrowth Analysis

PC12 pheochromocytoma cells were cultured in Dulbecco’s modified eagle’s medium (DMEM, Gibco–cat# 31966-021, Germany) at 37 °C and 5% CO_2_ using standard sterile cell culture techniques and seeded at a density of 2500 cells per well in a poly-d-lysine (Sigma)-coated glass-bottom 24-well plate. These were left overnight to attach, and the media was supplemented with either NGF in PBS to create a final concentration of 50 ng/mL (positive control) or an equal volume of PBS (therefore 0 ng/mL of NGF, negative control), 1 mg of cryogels loaded with 1000 ng/mL of NGF, or 1 mg of cryogels that were not loaded with NGF (*n* = 3 for each group). The cells were imaged by light microscopy (Olympus IX73) and then incubated at 37 °C, 5% CO_2_ for 5 days. The high concentration of 50 ng/mL (positive control) was used as a well-validated concentration for causing PC12 cell neurite outgrowth.^49^

After the incubation, the cells were once again imaged by light microscopy and then washed carefully (so as not to remove the cryogels from the well) and fixed with 4% formaldehyde for 10 min. The cells were permeabilized with 1% Triton x-100 in PBS with 3% goat serum (60 mg/mL, Jackson Immunoresearch, U.K.), 0.1% HEPES (1M, Gibco). This solution was removed and replaced with anti-βIII-tubulin antibody (raised in rabbit, diluted 1:1000 in blocking solution – BioLegend, #802001, Germany) and incubated at 4 °C overnight. The samples were then washed three times with PBS and incubated for 3 h with a goat anti-rabbit secondary antibody fluorescently labeled with Alexa 488 (Life Technologies #A11008, Germany) at a 1:200 dilution in blocking solution. During the last 5 min of incubation, the Hoechst nucleic acid (nuclear) stain was added at a dilution of 1:1000. The samples were then washed three times with PBS and imaged by means of the Dragonfly SPCLM using the 405, 488, and 637 nm laser diodes. A total of 20 images were taken per group from the three well replicates, and the lengths of the longest neurites were measured^50^ (up to a total of 100 cells) using the NeuronJ plugin for ImageJ (NIH software).

## Supporting Information Available

Additional data including template characterization, cryogel characterization, images of injection, and quantification of NGF release as a percentage of theoretical maximum (PDF)
