# Supplementary material for: Injectable Glycosaminoglycan-Based Cryogels from Well-Defined Microscale Templates for Local Growth Factor Delivery
Source: ACS Chem Neurosci. 2021 Mar 23;12(7):1178–88. doi: 10.1021/acschemneuro.1c00005 (PMC8033563; doi:10.1021/acschemneuro.1c00005)
Supplement: Supplementary file 1 — cn1c00005_si_001.pdf [file cn1c00005_si_001.pdf]

Supporting Information

Injectable Glycosaminoglycan Based Cryogels from Well-defined Microscale Templates for Local Growth Factor Delivery

Ben Newland[1,2*], Heike Newland[1], Francesca Lorenzi[1, 3], Dimitri Eigel[1], Petra B. Welzel[1], Dieter Fischer[1], Wenxin Wang[4], Uwe Freudenberg[1], Anne Rosser[5,6], Carsten Werner[1].

[1] Leibniz-Institut für Polymerforschung Dresden e.V., Max Bergmann Center of Biomaterials Dresden, Hohe Straße 6, D-01069 Dresden, Germany.
*Corresponding Author Email: newlandb@cardiff.ac.uk
[2] School of Pharmacy and Pharmaceutical Sciences, Cardiff University, King Edward VII Avenue, Cardiff, CF10 3NB, U.K.
[3] Dipartimento di Scienze Chimiche, Università degli Studi di Padova, via Francesco Marzolo, 1
35131- Padova, Italy
[4] Charles Institute for Dermatology, University College Dublin, Dublin, Ireland
[5] Brain Repair Group, School of Biosciences, Cardiff University, Cardiff, U.K.
[6] Brain Repair And Intracranial Neurotherapeutics (BRAIN) Unit, Neuroscience and Mental Health Research Institute, Cardiff University, Hadyn Ellis Building, Maindy Road, Cardiff. CF24 4HQ3

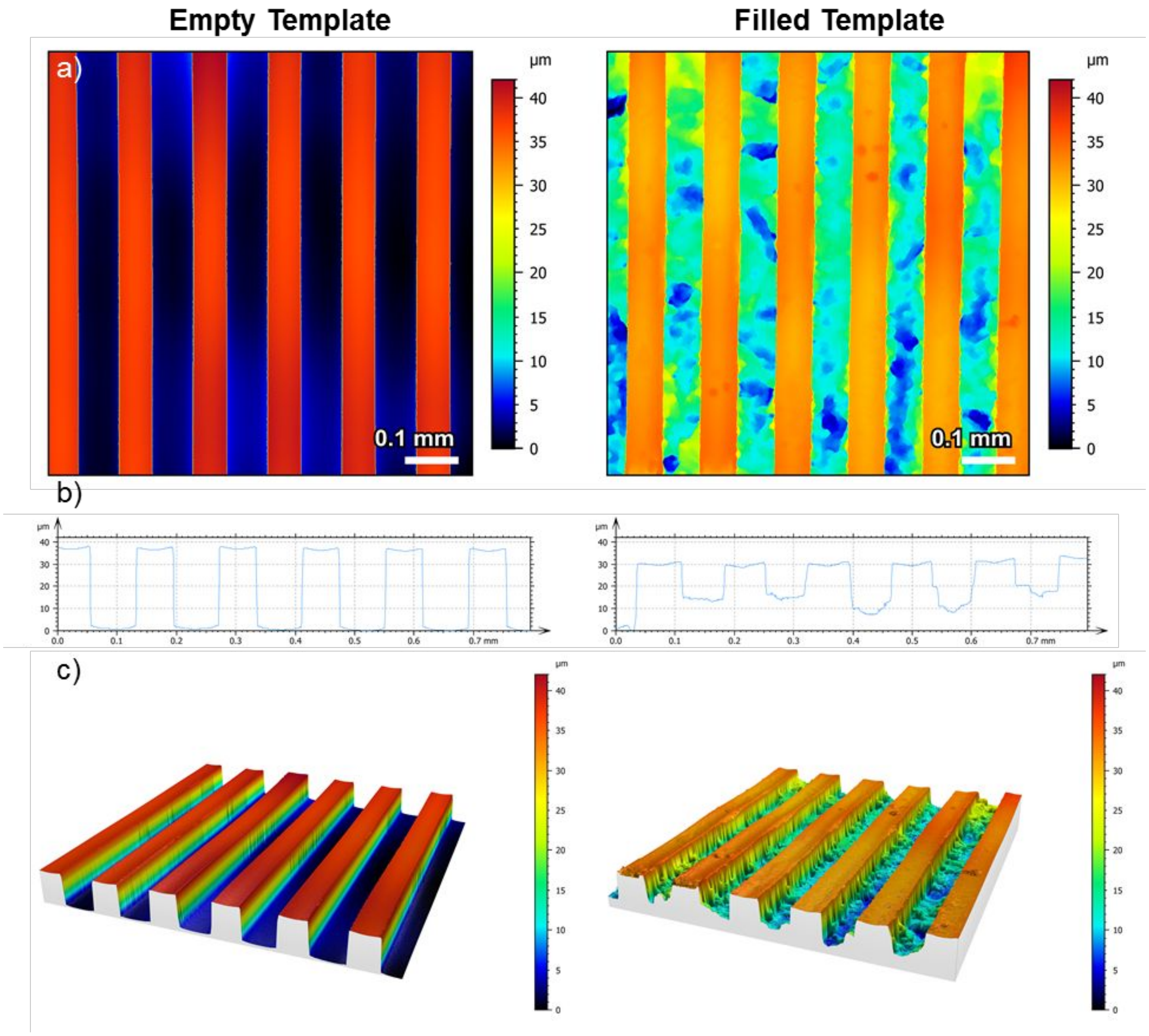

Figure S1 – showing template characterization of the 70µm wide channels via multi-pinhole confocal microscopy (NanoFocus) using µsoft metrology software. Both empty (left hand side) and filled (right hand side) template were analyzed showing the surface view (a), profile measurement (b) and a 3D render of the template (c).

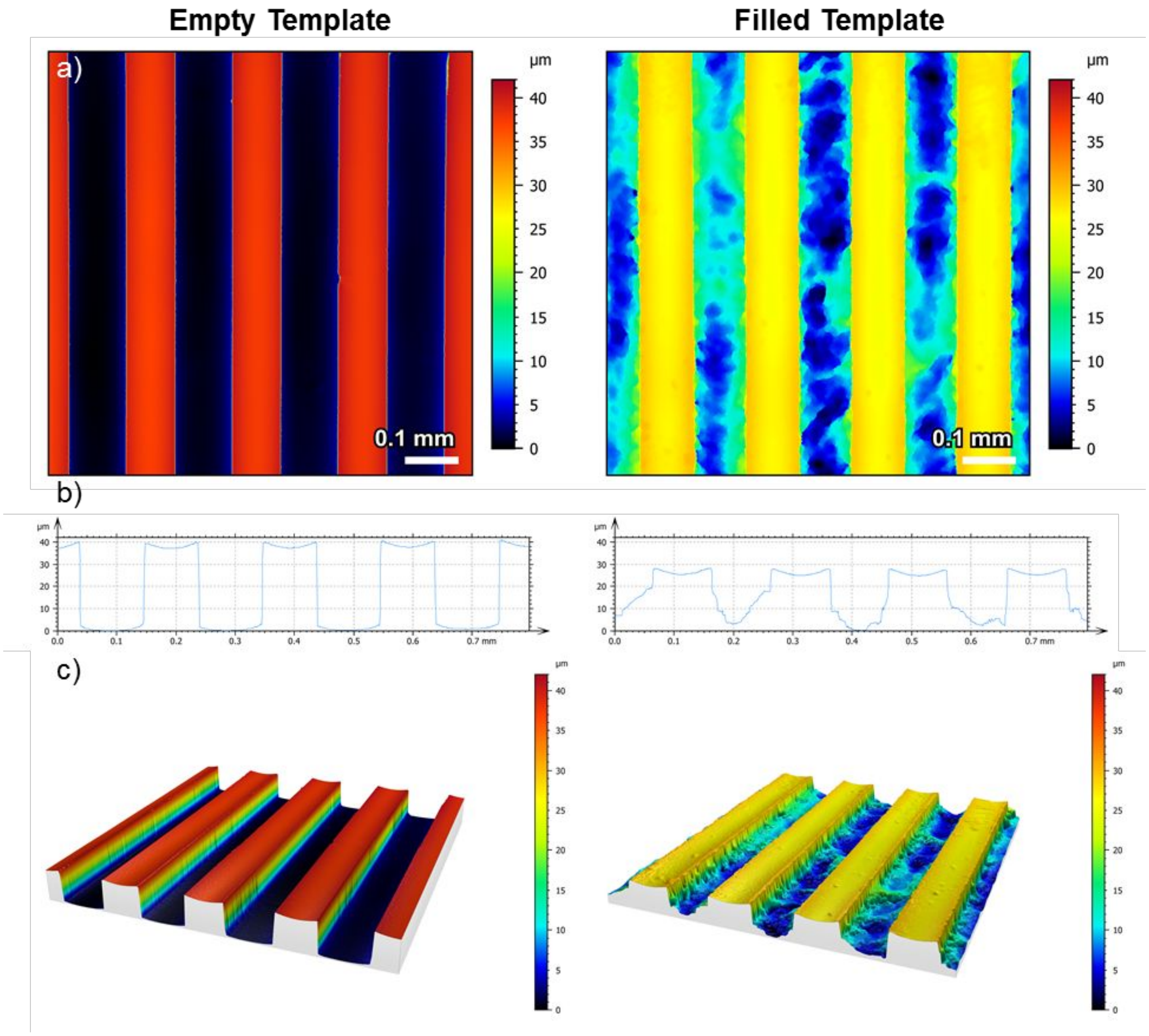

Figure S2 – as above, showing template characterization of the 100μm wide channels both empty (left hand side) and filled (right hand side).

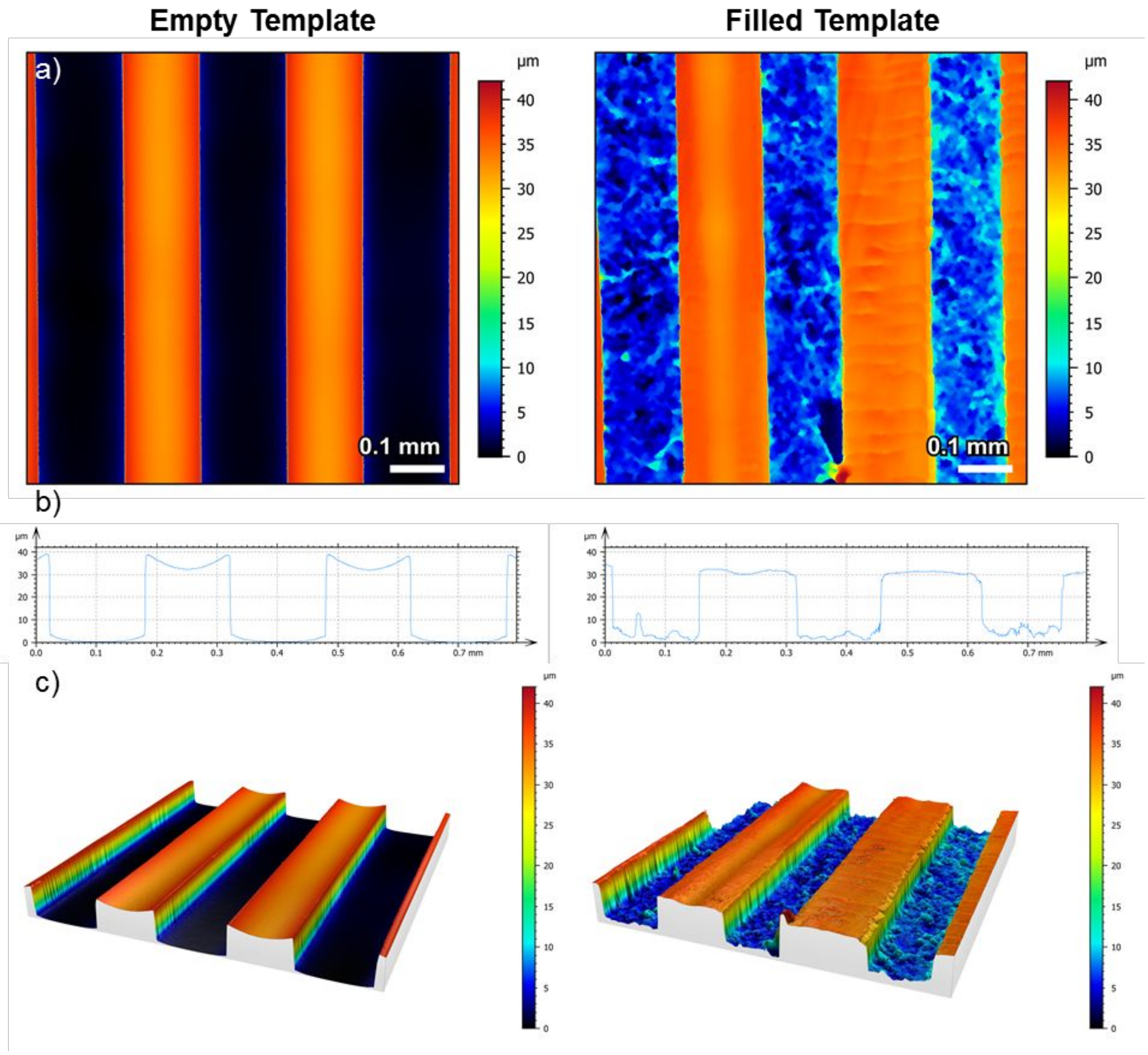

Figure S3 – showing template characterization of the template with largest channel size (150µm wide channels) both empty (left hand side) and filled (right hand side).

Comparison of all 4 spectra (660 – 1850 cm-1), normalised

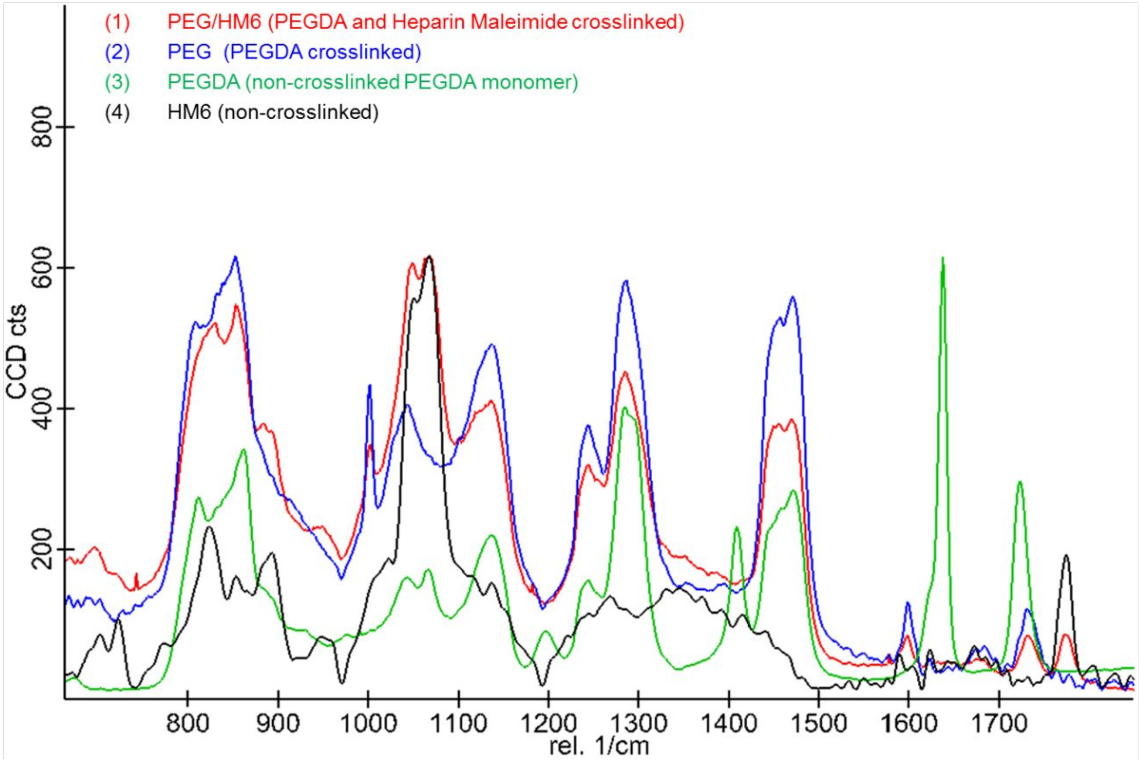

Figure S4 – Raman spectroscopy analysis of the non-crosslinked PEGDA monomer (green), the maleimide modified heparin (black), a sample of crosslinked PEGDA (without heparin, blue) and the PEGDA/heparin cryogels (red).

Comparison of all 4 spectra (1550 – 1850 cm-1)

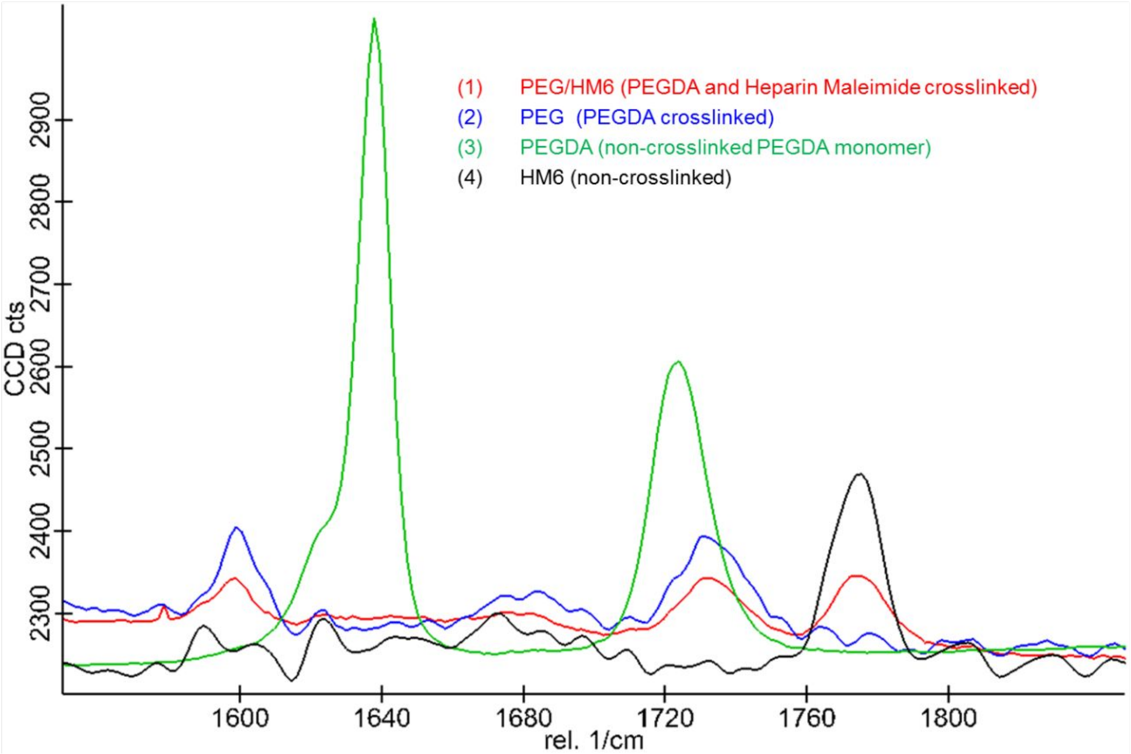

Figure S5 – Raman spectroscopy analysis of the non-crosslinked PEGDA monomer (green), the maleimide modified heparin (black), a sample of crosslinked PEGDA (without heparin, blue) and the PEGDA/heparin cryogels (red) showing the reduction in the peak at 1640 cm^{-1} and 1774 cm^{-1} (green/black to red) due to the reaction of the maleimide with the acrylate.

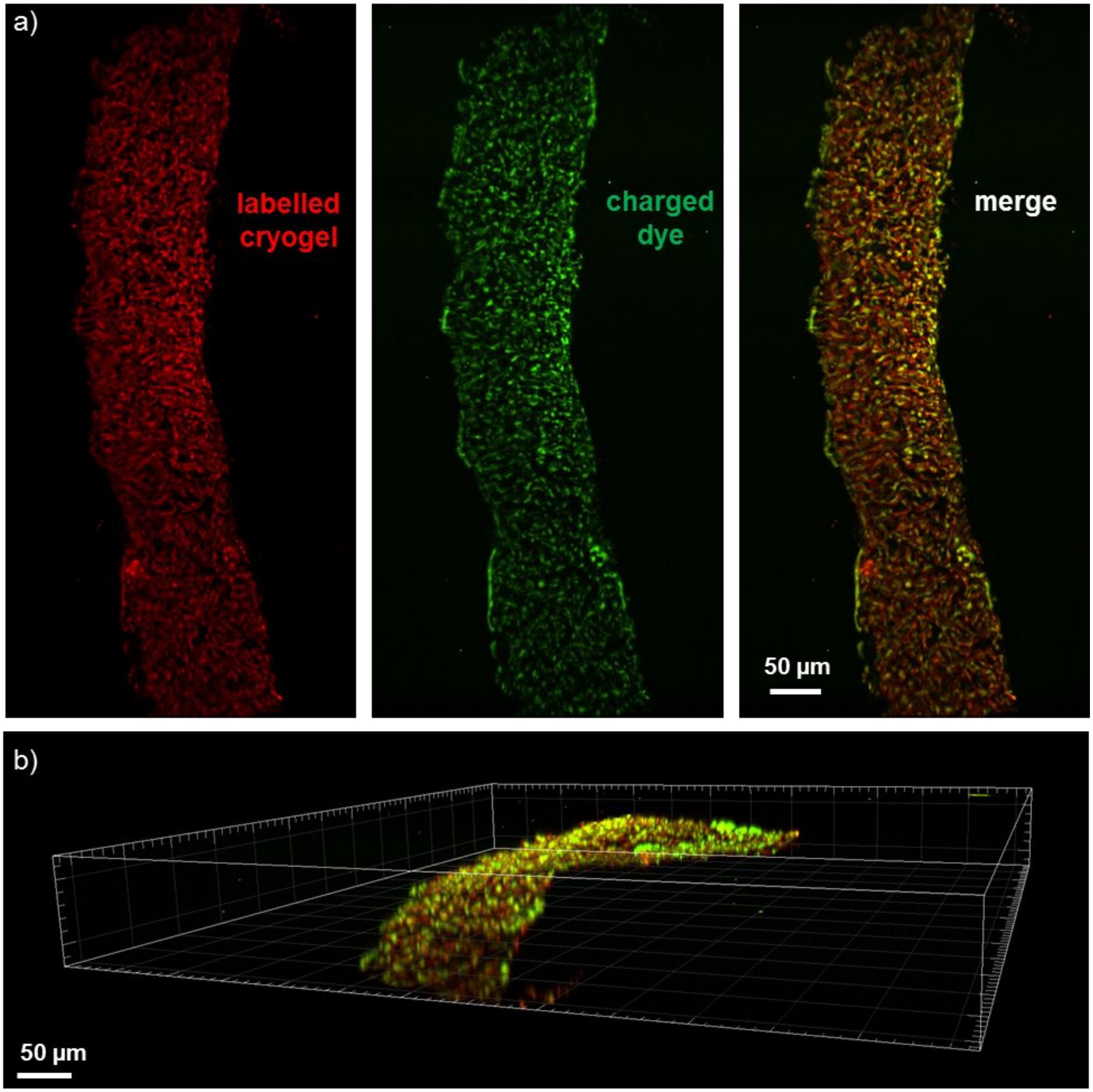

Figure S6 – (a) confocal laser fluorescence microscopy of an ATTO 647 labelled cryogel (red) that had been submersed in a solution of the positively charged, fluorescent doxorubicin molecule (green). Since doxorubicin binds to heparin, this method was used to indirectly show the distribution of heparin throughout the cryogel structure (merge panel). The same cryogel image shown in three dimensions (b) also confirms an even distribution of heparin throughout the cryogel, though there are some areas with more green intensity (indicating more heparin) and areas of more red intensity (indicating less heparin).

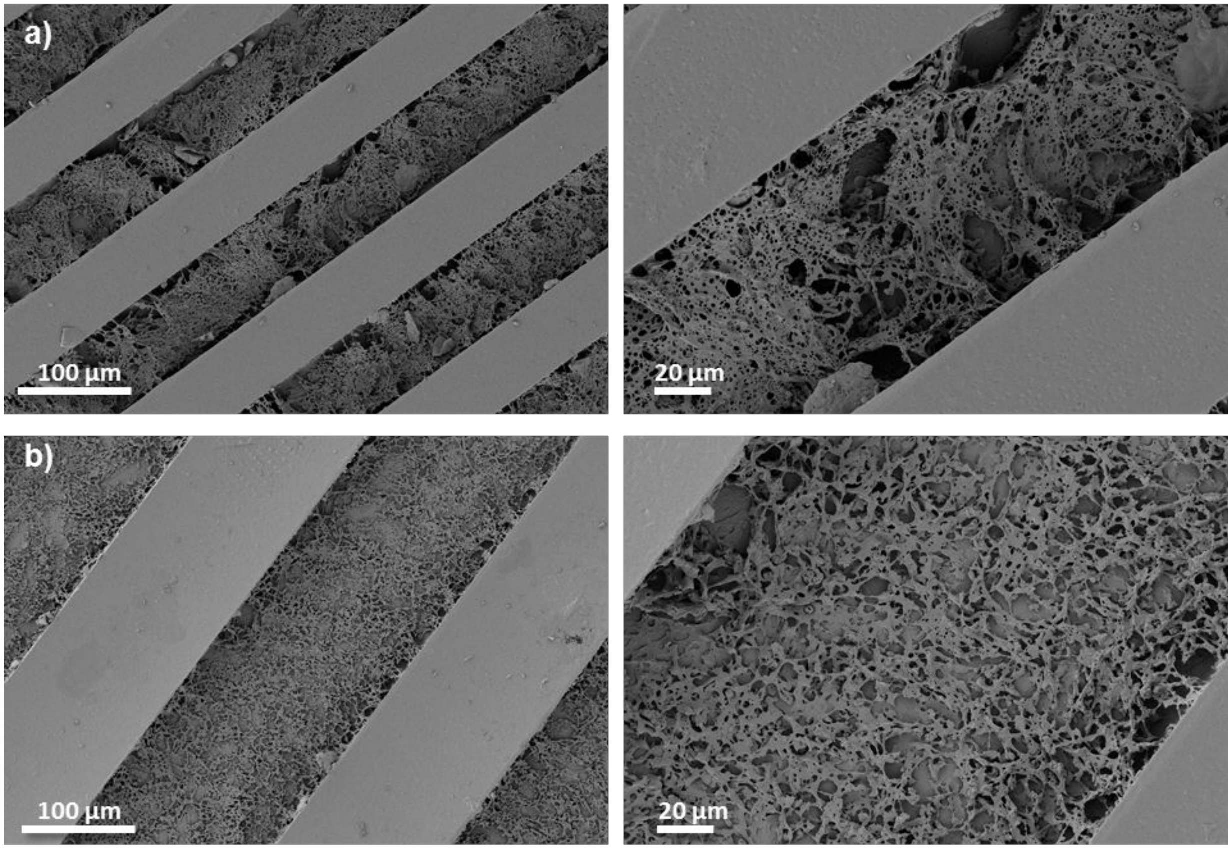

Figure S7 – Scanning electron microscopy analysis of cryogel filled templates with a channel width of 70 µm (a) and 150 µm (b).

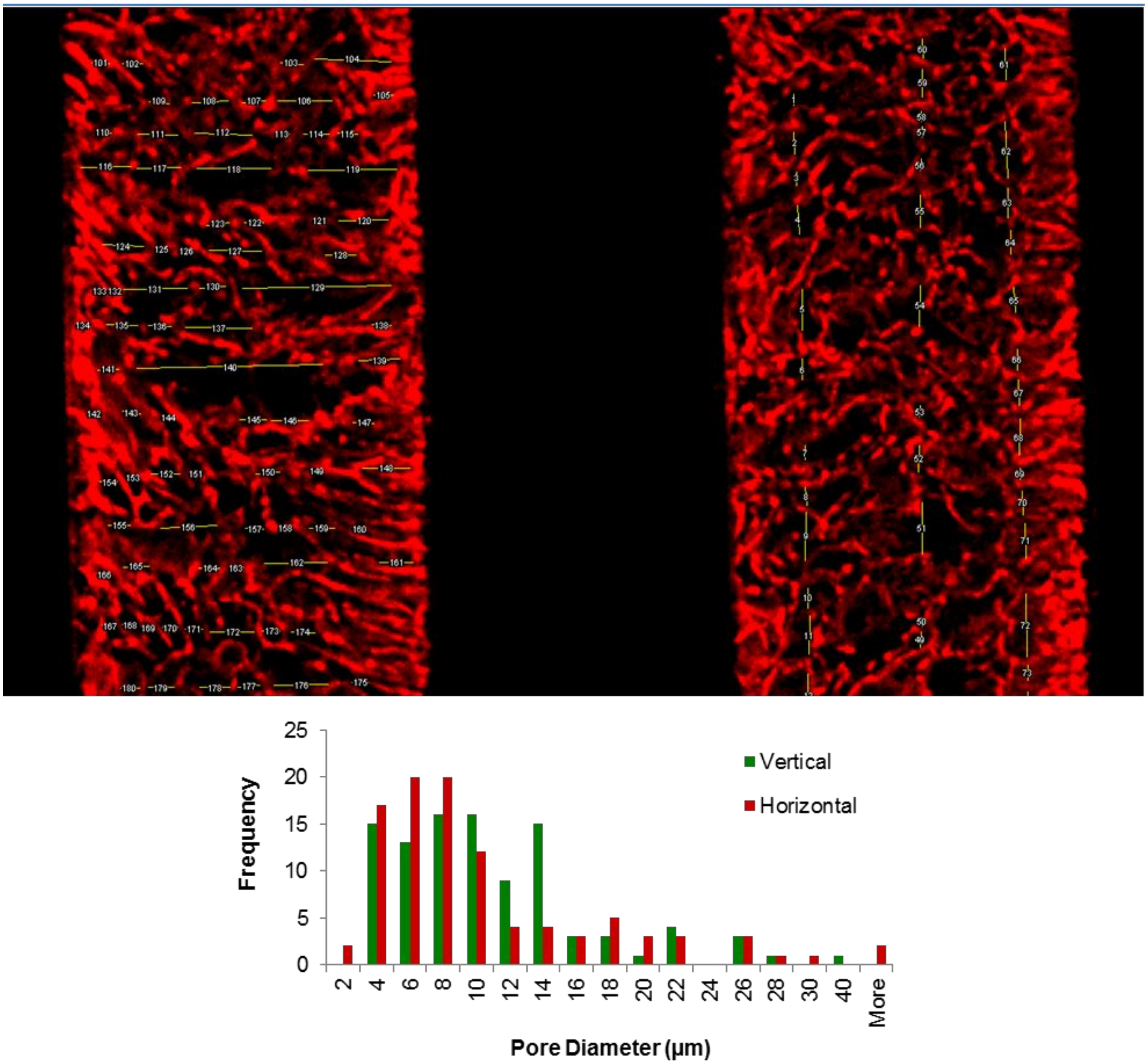

Figure S8 – Pore size analysis, showing an example image of the measurements made using ImageJ software, and the subsequent pore diameter distribution below. The average pore size was 9.9 µm when an average of the vertical and horizontal measurements was made.

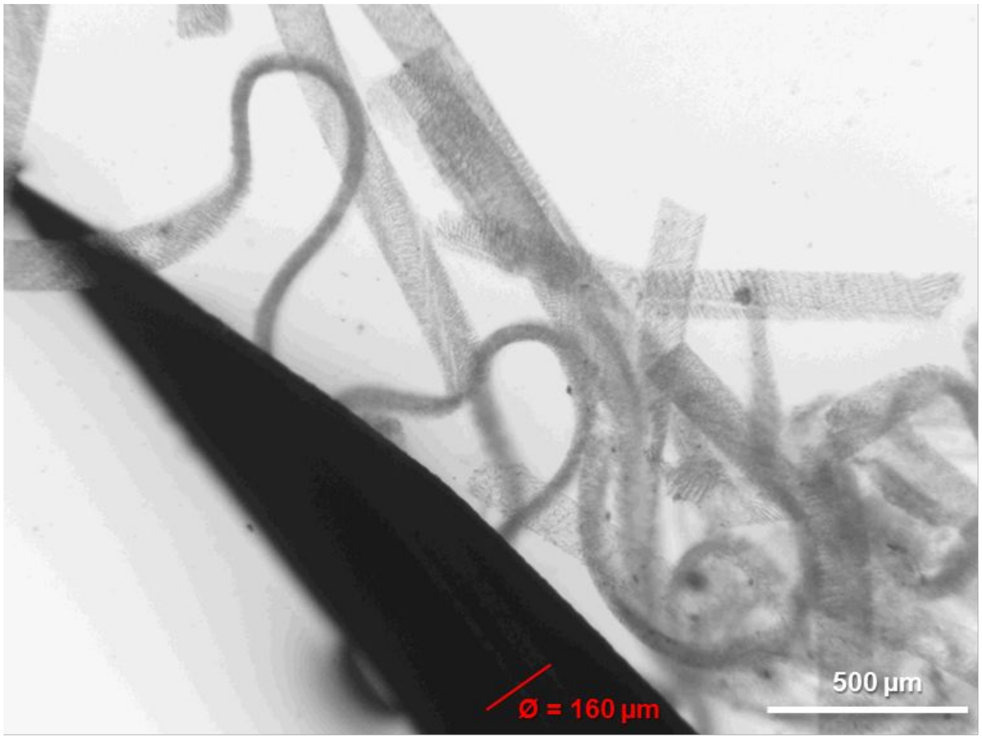

Figure S9 – A light microscope image taken after cryogel scaffolds (100 µm diameter) were injected out of a 30 gauge needle (inner diameter represented to scale).

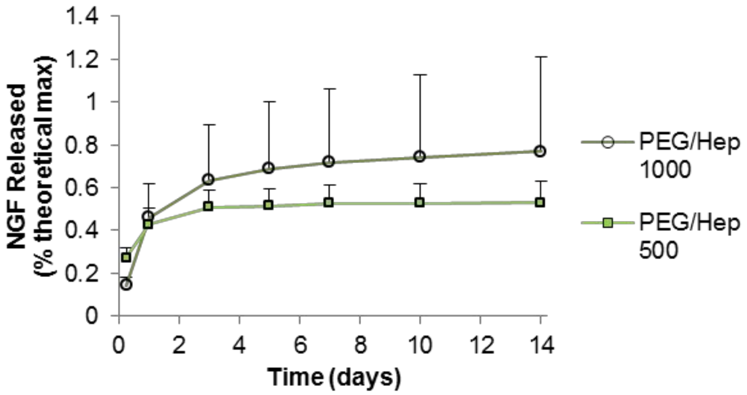

Figure S10 – nerve growth factor release as a percentage of the total growth factor loaded to the cryogel scaffold showing that only a small fraction of the growth factor is released (n=3) (error bars represent the cumulative value of standard deviation).
